# Diversity, Phylogeny and Antagonistic Activity of Fungal Endophytes Associated with Endemic Species of *Cycas* (Cycadales) in China

**DOI:** 10.3390/jof7070572

**Published:** 2021-07-18

**Authors:** Melissa H. Pecundo, Thomas Edison E. dela Cruz, Tao Chen, Kin Israel Notarte, Hai Ren, Nan Li

**Affiliations:** 1 South China Botanical Garden, Chinese Academy of Sciences, Guangzhou 510650, China; melissa.pecundo@gmail.com (M.H.P.); renhai@scib.ac.cn (H.R.); 2 Fairy Lake Botanical Garden, Chinese Academy of Sciences, Shenzhen 518004, China; taochen@szbg.ac.cn; 3 University of Chinese Academy of Sciences, Beijing 100049, China; 4 Department of Biological Sciences, College of Science, University of Santo Tomas, Manila 1008, Philippines; tedelacruz@ust.edu.ph; 5 Fungal Biodiversity, Ecogenomics and Systematics (FBeS) Group, Research Center for the Natural and Applied Sciences, University of Santo Tomas, Manila 1008, Philippines; kinotarte@gmail.com

**Keywords:** antifungal activity, co-cultivation assay, coralloid roots, fungal diversity, host habitat

## Abstract

The culture-based approach was used to characterize the fungal endophytes associated with the coralloid roots of the endemic *Cycas debaoensis* and *Cycas fairylakea* from various population sites in China. We aim to determine if the assemblages of fungal endophytes inside these endemic plant hosts are distinct and could be explored for bioprospecting. The isolation method yielded a total of 284 culturable fungal strains. Identification based on the analysis of the internal transcribed spacer (ITS) rDNA showed that they belonged to two phyla, five classes, eight orders and 22 families. At least 33 known genera and 62 different species were confirmed based on >97% ITS sequence similarity. The most frequent and observed core taxa in the two host species regardless of their population origin were *Talaromyces*, *Penicillium*, *Fusarium*, *Pochonia* and *Gliocladiopsis.* Seventy percent was a rare component of the fungal communities with only one or two recorded isolates. Contrary to common notions, diversity and fungal richness were significantly higher in *C. debaoensis* and *C. fairylakea* collected from a botanical garden, while the lowest was observed in *C. debaoensis* from a natural habitat; this provides evidence that garden management, and to a minor extent, ex-situ conservation practice, could influence fungal endophyte communities. We further selected nineteen fungal isolates and screened for their antagonistic activities via a co-cultivation approach against the phytopathogens, *Diaporthe* sp. and *Colletotrichum* sp. Among these, five isolates with high ITS similarity matches with *Hypoxylon vinosupulvinatum* (GD019, 99.61%), *Penicillium* sp. (BD022, 100%), *Penicillifer diparietisporus* (GD008, 99.46%), *Clonostachys rogersoniana* (BF024, 99.46%) and *C. rosea* (BF011, 99.1%), which showed exceptional antagonistic activities against the phytopathogenic fungi with a significant inhibition rate of 70–80%. Taken together, our data presented the first and most comprehensive molecular work on culturable fungal endophytes associated with the coralloid roots of cycads. Our study also demonstrated that about 5% of fungal endophytes were not detected by the high-throughput sequencing approach, implying the equal importance of a culture-dependent approach to study fungal communities of cycads. We further highlighted the potential role of endemic and rare plants to discover and isolate unique plant-associated fungal taxa with excellent biocontrol properties.

## 1. Introduction

Complex and heterogeneous assemblages of fungal communities that do not elicit symptoms of disease are hidden intracellularly in the internal tissues of plants [1,2]. They are termed as fungal endophytes as they spend part or the entirety of their life cycle inside healthy tissues of plants [1]. Fungal endophytes also represent an important component of the root microbiota and provide benefits to their hosts by acting as biocontrol agents, plant growth promoters, environmental stress regulators, and host protectors [3,4]. Root endophytic fungi were reported in different plants across a wide range of environments. For instance, mounting evidence showed a highly diverse species of root endophytes in plants thriving in hostile environments, such as deserts [5,6], salt marshes [7,8], arid and geothermal environments [9,10] and karst rocks [11]. The functional symbiosis or the establishment of mutualistic relationships between root fungal endophytes with their hosts are crucial for their survival and adaptation to these extremophilic environments [11].

The diversity and community assemblages of fungal endophytes are said to be filtered by a multitude of factors. The interactions that exist between them were reported to be influenced by both environmental and physiological factors resulting in a balance and beneficial relations between parties [12]. For instance, previous studies demonstrated that soil properties, nutrient availability, root exudates and traits and local climatic conditions, e.g., temperature and precipitation, are vital drivers that structure endophytic microfungal communities in plant roots [13,14,15,16,17,18]. Geographical distance also has a significant effect on fungal communities where plant hosts occupying the same geographic region with nearly similar climatic conditions result in similar endophytic fungal communities [17]. In some studies, these abiotic factors are coupled with host-related factors such as genetic differences among inter/intraspecific hosts and influence the entire plant-fungal association [18,19]. This is particularly interesting to investigate especially since some plants belonging to a single species revealed both genetic distinctness and high dissimilar fungal communities [19].

Fungal endophytes recovered from a wide lineage of plants have been investigated for bioprospecting [2,20,21,22,23]. Particularly on gymnosperms, early studies have reported the production of secondary metabolites with strong antimicrobial activity [24,25]. Interestingly, multiple studies in the past showed that endophytic fungal communities associated with native and endemic plants may have the greatest promise for exploitation as they have the potential to host rare and unique assemblage of fungal communities. Some of the fungal endophytes that were isolated from native or endemic plants exemplarily produced antioxidant, anticancer and antimicrobial compounds [21,25,26,27,28,29]. This reflects the additional need to particularly investigate fungal communities associated with endemic host plants.

*Cycas* is the sole genus of the cycad family Cycadaceae [30], with several members endemic in one geographical region. Of the 120 accepted species, *Cycas debaoensis* Y.C. Zhong and C.J. Chen and *Cycas fairylakea* D.Y. Wang are rare, critically endangered and endemic to China [30,31]. Their natural populations were also found in different localities with varying climatic conditions. *C. debaoensis* was distributed at the open karst areas near the border of Guangxi and Yunnan provinces, while *C. fairylakea* grew naturally in moist closed forests in the East Guangdong province [30,31]. Environmental conditions with extreme drought and poor nutrients are the characteristics of the extant cycads’ habitats millions of years ago [32]. Cycads are then believed to evolve a differentiated organ called coralloid roots, which are morphologically distinguished by dichotomous branching, coral-like forms and apogeotropic growth [32]. These special roots were found to shelter a diverse group of endosymbiotic microorganisms. In fact, it is most recognized with its relationship to the symbiotic cyanobacteria manifesting a macroscopically visible green zone around its cortex [32]. An increase in attention relative to genomic research and conservation studies is particularly given for *Cycas* due to its rarity, taxonomic and evolutionary uniqueness, high degree of endangerment and extinction risk [33,34,35].

Microbial studies carried out on coralloid roots were relatively focused on either the root-cyanobacterial association [32] or the overall estimation of microbial diversity based on high-throughput sequencing [36,37,38,39]. Recently, a functional-based metagenomic study revealed that the coralloid roots of some *Cycas* spp. particularly contained microbes responsible for metal homeostasis, carbon metabolism and stress responses [40]. In this context, studies on culturable microbes, such as fungal endophytes from endemic cycads, merit equal importance as they could be explored for biodiscovery and the bioprospecting of bioactive metabolites. Therefore, the present study investigated the assemblages of culturable fungal endophytes in the coralloid roots of *C. debaoensis* and *C. fairylakea* collected from different sites in China. The plants are distributed in exclusively small geographical locations and are therefore expected to contain host-specific endophytes. Specifically, this study aims to (1) identify the diversity, phylogeny and taxonomic composition of fungal endophytes associated with the coralloid roots of *C. debaoensis* and *C. fairylakea*; (2) to examine whether or not the hosts’ identity, population or sampling sites, i.e., at local and regional scales, and transplantation influence the pattern of diversity and community composition of fungal endophytes; and (3) screen for the antagonistic potential of the selected fungal endophytes against phytopathogens for biocontrol and bioprospecting.

## 2. Materials and Methods

### 2.1. Sampling Site Descriptions and Sample Collection

**Figure 1:**
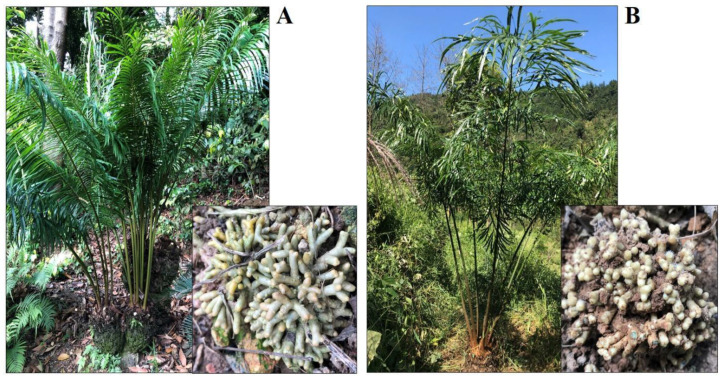
Host species of *Cycas* and the appearance of their coralloid roots growing above the surface of the soil. (**A**) *Cycas fairylakea* from the ex-situ conservation site in National Cycad Germplasm Conservation Center, Fairy Lake Botanical Garden, Guangdong, and (**B**) *Cycas debaoensis* from the reintroduction site in Debao County, Guangxi.

Samples of coralloid roots were taken from the two host species, *C. debaoensis* and *C. fairylakea* (Figure 1) from different population or sampling sites, i.e., an ex-situ conservation botanic garden, a natural habitat and a reintroduction site where propagated populations from a plant nursery in a botanical garden were reintroduced to the natural habitat. These propagated populations were derived from seeds collected in their natural habitat and grown in plant nurseries in a botanical garden before re-planting in the reintroduction site. The sampling sites are located in the Guangxi and Guangdong provinces in China. Details of the host species and sampling localities are provided in Table 1. The sampling sites in the Guangxi province possess a distance of 1100 km from the sampling sites in the Guangdong province. The former region in South China has a mountainous terrain and is home to spectacular karst landforms and natural reserves. The latter region facing the South China Sea is diverse and intersected by several basins with scattered alluvial valleys. Both regions have subtropical to tropical climate. In Guangxi, the summers are generally long, hot and humid lasting from April to October. The average annual temperature ranges from 17 °C to 23 °C and the annual precipitation ranges from 1080 mm to 1730 mm. In the Guangdong province, a more humid climatic condition marked by high temperature and abundant rainfall, especially between the months of April to June, is experienced. It also has a prolonged hot but wet summer with an annual rainfall of 1400–2000 mm. The average annual temperature is 22 °C. Six plant individuals with thick and healthy coralloid roots were selected per population site. From each plant host, two to three clusters of roots with a small amount of soil were collected and placed inside clean Ziplock bags, kept in an ice chest, brought to the laboratory and immediately refrigerated at 4 °C until further processing.

### 2.2. Coralloid Root Processing and Fungal Endophyte Isolation

The coralloid roots were cut into small clusters, washed under running tap water and gently scrubbed with a soft bristle brush to remove adhering soil and other debris. Under sterile conditions, the roots were first rinsed in cold milliQ water and the root surface was chemically treated according to the method described [15], with some modifications: sodium hypochlorite (NaOCl, ~2% active chlorine) for five minutes, 70% ethyl alcohol for five minutes and sterile milliQ water four times for 1–2 min each time. The roots were then blot-dried using sterile paper towels and aseptically dissected into thin longitudinal segments with approximate uniform sizes (~5 cm long, ~2 mm thick) using sterile blades. The root segments were then plated on a half-strength Malt Extract Agar (MEA, BD Difco, BD Shanghai Co., Ltd., Shanghai, China) amended with 400 µg ml^−1^ antibiotic streptomycin to prevent bacterial contamination. A total of 36 root segments were cut and placed on six petri dishes per host individual. Overall, a total of 1080 root segments, 648 from *C. debaoensis* (6 plant individuals × 36 root segments × host species × 3 sites) and 432 from *C. fairylakea* (6 plant individuals × 36 root segments × host species × 2 sites), were utilized in this study. Six root segments were equidistantly placed onto the surface of each plated medium using sterile forceps. Sterility control plates were also prepared by plating water from the final wash and imprinting the roots before dissection into segments onto separate and clean MEA with antibiotic. To rule out the possible effect of air contamination, two clean MEA plates were left open while performing the experiment. The isolation and control plates were then incubated in a growth chamber at 28 ± 2 °C. Incubation and checking of plates were performed up to 4 weeks to allow the isolation of slow growing fungi. The absence of fungal colony growth in the sterility control plates indicates a successful and efficient surface sterilization [41]. Observation of the culture plates was conducted every day to observe emerging hypha from the edge of the coralloid roots. Isolation was immediately performed by cutting a small portion on the edge of fungal hypha and transferring it to a freshly prepared medium but without antibiotics. Purification of fungal endophytes was carried out using hyphal/spore tip method. All isolates were cultured and maintained in agar slants at the Cycad Symbiotic Microorganism Lab (CSML) in the Research Building of the Fairy Lake Botanical Garden, Shenzhen.

### 2.3. Morphocultural Characterization and Morphotype Groupings of Fungal Endophytes

After successful isolation and purification of fungal endophytes, all isolates were grown on Potato Dextrose Agar (PDA, BD Difco, BD Shanghai Co., Ltd.). The axenic isolates were then observed and grouped according to their distinct colonial features and assigned to a specific group code. The morphological characteristics that were considered for morphotype groupings were the colony appearance, color, form, elevation and margin. The color and appearance of the colony on top and below (reverse) were also noted [41]. Representatives from each morphotype groups were subsequently grown for DNA extraction and molecular identification.

### 2.4. DNA Extraction, PCR Amplification and ITS Gene Sequencing

Genomic DNA was extracted from a 7–10 day old fungal culture representing 80 morphospecies following the modified procedure [42]. Approximately 100 mg of fresh fungal mycelium was used and ground in sterile mortar and pestle with liquid nitrogen. The crushed fungal mycelium was then put in a 2 mL sterilized microcentrifuge tube with 60 mg sterile glass bead and 700 µL of extraction buffer with the following composition: 100 mM Tris HCl (pH 8.0); 50 mM EDTA, 3% SDS. Homogenization was performed once on a tissue homogenizer (Tissuelyser 24, Jingxin Tech., Yiyang, China) at 6 m/s for 30 s. Centrifugation of the fungal tissue homogenate was then carried out for 10 min at 12,000 rpm. Approximately 600 uL of supernatant was then transferred to a new microcentrifuge tube and treated with 2 uL of RNase A (10 mg/mL). DNA extraction was performed twice by putting equal volume of PCI (Phenol Chloroform Isoamylol: 25:24:1). Precipitation was performed with chilled absolute ethyl alcohol at −20 °C for 1 h. The DNA pellet was washed twice with 70% ethyl alcohol, air dried and dissolved in 1× TE (pH 8.0) buffer. The ribosomal DNA internal transcribed spacer (ITS) region (ITS1–5.8S–ITS2) was amplified using the forward primer ITS1 (5′-TCCGTAGGTGAACCTGCGG-3′) and reverse primer ITS4 (5′-TCCTCCGCTTATTGATATGC-3′) [43]. Following a previous study [44], the final reaction volume was 25 µL containing 2.5 µL 10× PCR (Mg^2+^free) buffer, 2 µL of 25 mM MgCl_2_ (Takara), 1.5 µL dNTP mixture (2.5 mM), 1 µL of each primer (10 pmoles), 0.2 µL (5 U/mL) of Taq polymerase, 1.5 µL template DNA and 15.3 µL sterile dH_2_O. PCR amplification was carried out on a BIO-RAD T100 Thermal Cycler with initial denaturation for 5 min at 94 °C, followed by 35 cycles (35 s at 94 °C, 50 s at 52 °C, 1 min at 72 °C) and a final extension of 5 min at 72 °C. The quality of PCR products was then checked on 0.8% agarose gel in 1× TAE buffer. The amplicons were sent to TSINGKE Biotechnology Co., Ltd. (Guangdong, China) and sequenced in both directions using the abovementioned primers on ABI 3730 Automated Sequencer.

### 2.5. Molecular Identification and Phylogenetic Analysis of Fungal Endophytes

A total of 120 fungal sequences were generated from this study in which the 119 sequences were deposited to the National Center for Biotechnology Information (NCBI) Taxonomy Database (http://www.ncbi.nlm.nih.gov/taxonomy) GenBank with accession numbers MW750556, MW760722–MW760840. We then compared and identified our sequences with the data deposited in NCBI databases via the BLAST algorithm and downloaded the closest match sequences (≥97% identity). Alignment of the sequences was performed using the Auto algorithm of MAFFT v7.450 [45] and manually edited in Geneious Prime 2020.2 [46]. Phylogenetic reconstruction was then performed with the Maximum Likelihood (ML) topological method. The best fit model used for the ITS sequence alignment were the general time reversible and gamma distributed with invariant sites (GTR+G+I) as determined by the Bayesian information criterion (BIC) in the model selection tab of IQ-TREE v6.10 (iqtree.cibiv.univie.ac.at) [47]. Bootstrap analyses and calculations were based on 1000 replicates. The ML was run on IQ-TREE v6.10 [47] and the resulting phylogenetic tree consensus file was viewed in FigTree v1.4.3, rooted with *Mucor circinelloides* (phylum Mucoromycota). Accession numbers of sequences retrieved from GenBank are also included in our phylogenetic tree.

### 2.6. Statistical and Diversity Analysis

The colonization rate (CR) of fungal endophytes was determined as the total number of isolates recovered from a given site divided by the total number of root segments prepared in that particular site multiplied by 100. The isolation frequency (IF) was also computed by the number of isolates recovered from a given site divided by the overall number of isolates multiplied by 100. In addition, comparisons of the fungal endophyte composition and diversity between the host species (*C. debaoensis* vs. *C. fairylakea*) and populations sites (botanical garden vs. reintroduction site vs. natural habitat) were calculated using the species richness (S), Shannon–Wiener (H’) and Fisher’s-Alpha (FA) diversity statistics. The number of species recovered from a particular population site or host species represented the species richness. The Shannon and Fisher’s alpha indices were computed using the relative proportion of fungal species in a specific population site and host species. The taxonomic diversity index through the computation of Species (S) over Genus (G) (S/G) ratio was also determined per host species and population sites. Moreover, the β-diversity using the non-metric multidimensional scaling (NMDS) based on Bray–Curtis dissimilarity matrices was performed using the datasets from population sites and host species. The difference in the diversity was statistically analyzed by performing the analysis of variance (*p* < 0.05) using Paleontological statistics software PAST (version 4.03) [48].

The datasets from our previous study [39], in which we reported fungal composition using high throughput sequencing, were also extracted and compared with the datasets obtained using the culture-dependent technique in the present study to provide insights on the overall community composition and structure of fungal endophytes associated with the cycads. We simply expressed the data as a Venn diagram to show whether we recovered those taxa that were recorded by culture-independent molecular methods.

### 2.7. In Vitro Co-Cultivation Assay between Fungal Endophytes and the Test Pathogens

Endophytic fungal cultures were screened to test their ability to suppress mycelial growth of fungal pathogens on a direct confrontation using the in vitro co-cultivation assay. Fungal co-cultivating technique is a quick and efficient method to mimic microbial interactions in their natural environment and allow the identification of microbes with antimicrobial activities [49,50,51,52]. In the present study, only fungal endophytes with distinct colonial characteristic features, e.g., pigmented colony and production of coloring/exudates on media, were subjected to antagonistic screening [52]. In addition, we selected the fungal endophytes based on recent published literatures and focused on isolates that were less-studied (very few existing studies on their activities were conducted), rare taxa (fungal endophytes with single isolate or were not commonly reported) and those that were reported to exhibit strong bioactivities against other microorganisms. Therefore, nineteen isolated fungal endophytes were examined. Meanwhile, the two fungal phytopathogens were recovered from the symptomatic-diseased leaf of *C. debaoensis* and were identified as *Diaporthe* sp. strain CdP01 and *Colletotrichum* sp. strain CdP02. Fungal discs (6 mm) were produced from the cultures of phytopathogens and the challenged root endophytic isolates. The fungal endophyte disc was placed on the center of the plate with half-strength PDA. The discs of the two phytopathogens were placed in the same manner in the same plate at a distance of approximately 3 cm from the endophyte. Control plates were also produced with only inoculated test phytopathogens in the absence of endophytic fungus. The assay was conducted with three biological replicates. Observations were carried out for ten days. The radial growths of the mycelia of the test pathogen on a control (r1) and in the direction of the endophytic fungus (r2) were measured and the percent inhibition in mycelial growth was calculated based on the formula I% = [(r1 − r2)/r1] × 100 [52]. Furthermore, the interactions of the fungal isolates during direct confrontations on co-cultivation assay were characterized based on the six types classification system [53]. These include the following: (1) Interaction type A: characterized by mutual intermingling growth of fungi with no visible sign of antagonism or inhibition of both fungi; (2) Type B: a mutual inhibition upon contact or at a distance between fungi with no to very minimal clear zone (usually <2 mm); (3) Type C: observed with inhibition of one fungus on contact with the inhibited species continued to grow at a significantly reduced rate, while the inhibitor species grew at a slightly, reduced rate or unchanged; (4) Type D: a mutual inhibition between fungi at a distance producing significantly clear zones of >2 mm; (5) Type E: inhibition of one fungus upon contact with another, the inhibitor species continuing to grow at a reduced rate through the inhibited colony; (6) Type F: inhibition of one species upon contact or at a distance and the inhibitor species continuing to grow at an unchanged rate through or over the inhibited fungus.

## 3. Results

### 3.1. Fungal Isolates Characterization and Taxonomic Assignment

**Figure 2:**
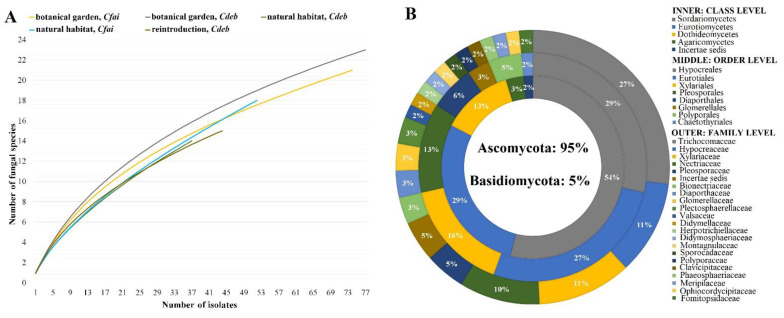
(**A**) Rarefaction curve showing the species richness of fungal endophytes plotted against the number of isolates; datasets are based on the host species (*Cycas debaoensis* and *Cycas fairylakea*) per their collection sites (botanical garden, natural habitat and reintroduction site). (**B**) Taxonomic distribution of the fungal taxa (*n* = 120) from the coralloid roots of the two endemic *Cycas* spp.

A total of 284 fungal endophytes were recovered from 1018 coralloid root segments examined from the two host species of *Cycas* (*n* = 30) collected in different population sites (Figure 2A). Overall, root samples from the host plants in the botanical garden yielded more isolates (IF) and significant higher colonization rates (CR) (IF = 27%, CR = 36% for *C. debaoensis*; IF = 26%, CR = 34% for *C. fairylakea*) than the samples from individuals in natural habitat (IF = 13%, CR = 17% for *C. debaoensis*; IF = 18%, CR = 24% for *C. fairylakea*) and from the reintroduced populations (IF = 15%, CR = 20% for *C. debaoensis*) (Table 2). *C. debaoensis* (IF = 56%, CR = 24%) had a higher isolation frequency and a lower colonization rate than *C. fairylakea* (IF = 44%, CR = 29%). Our isolates are composed of spore-forming species and mycelia sterilia on potato dextrose agar. Mycelia sterilia are fungal endophytes that failed to sporulate even after prolonged grown in culture media. They were observed based on their morphocultural characteristics, such as growth pattern, colony appearance, form, margin and color (front and reverse). The fungal isolates that share the same colonial features and obtained from the same host species and site were grouped together and considered to belong to the same morphotaxa. Overall, 120 isolates (42% of the total isolates) were maintained and subsequently sequenced for molecular identification and phylogenetic analysis. The high-quality ITS sequence data in combination with its cultural appearance were used to assign each isolate to a corresponding taxonomic unit. The closest match species and other information were identified on the BLASTn search (Supplementary Table S1). A similarity value of ≥97% was observed among sequenced fungal isolates except for GD016 with 95% percent identity against its nearest BLAST hit (Supplementary Table S1).

The majority of the fungal isolates belonged to Ascomycota (95% of the total isolates) and a few to Basidiomycota (5%) (Figure 2B). Furthermore, a total of five classes, eight orders and 22 families were identified. The fungal classification with the highest proportion was Sordariomycetes (54%), followed by Eurotiomycetes (29%) and Dothideomycetes (13%). A small proportion of isolates, with 3% and 2%, were classified as Agaricomycetes and as unclassified incertae sedis, respectively. At the family level, Trichocomaceae (27%) was the most diverse, followed by Hypocreaceae (11%), Xylariaceae (11%) and Nectriaceae (10%) (Figure 2B). The same number of isolates (5%) was classified into Pleosporaceae and as incertae sedis. The other families in the phylum Ascomycota were identified with ≤3% abundance (Figure 2B). Isolates in the phylum Basidiomycota were distributed into families of Fomitopsidaceae (3%), Polyporaceae (2%) and Meripilaceae (2%).

### 3.2. Phylogenetic Analysis of Fungal Endophytes Associated to Cycas debaoensis and Cycas fairylakea

**Figure 3:**
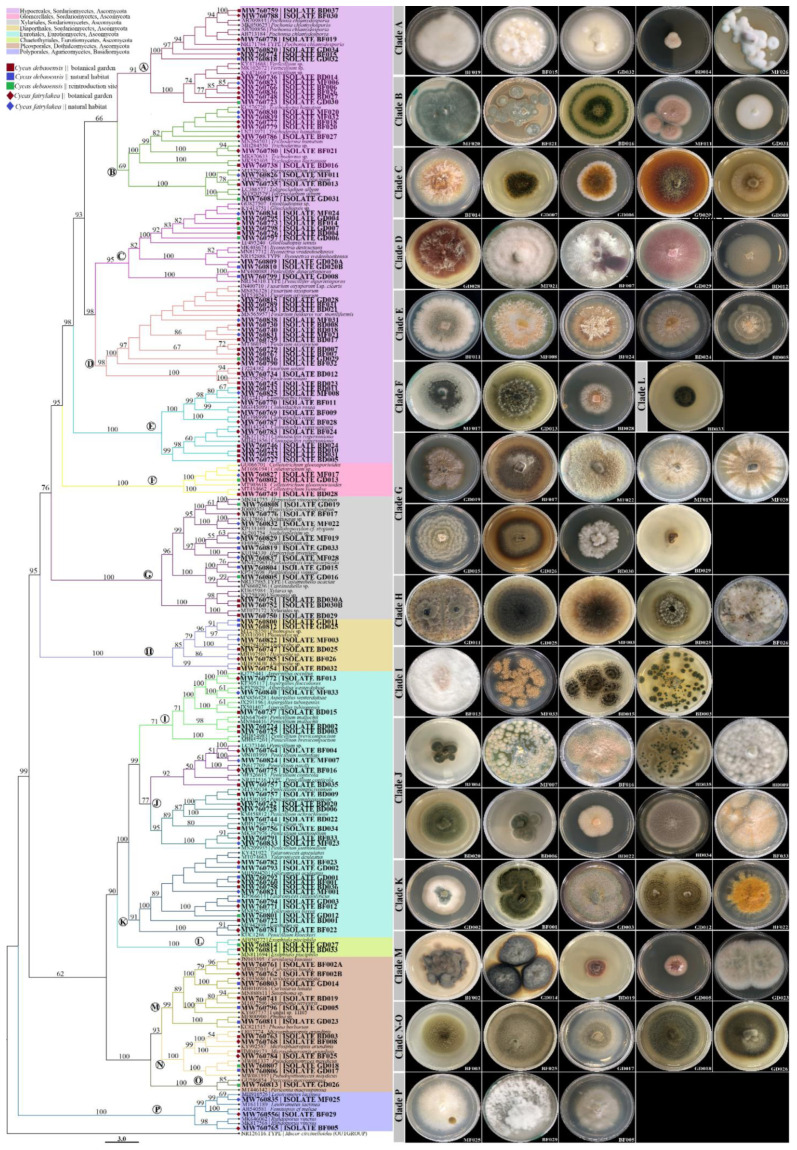
A maximum likelihood (ML) analysis showing the phylogenetic relationships of root fungal endophytes. The tree has a total of 233 fungal sequences, in which 119 fungal sequences came from the present study, 113 reference sequences of close relatives came from the NCBI database and the sequence from the type specimen of *Mucor circinelloides* (NR126116; phylum Mucoromycota; order Mucorales) as the outgroup. The bootstrap support values are indicated at the nodes. In bold font are the isolate code and sequence accession number of the fungal endophytes obtained from the coralloid roots of *Cycas debaoensis* and *Cycas fairylakea*. Fungal isolates are further highlighted in different colors according to the order classification. The tip shape of each isolate in the tree is a key in determining its source of host species and collection origin. Images on the right of the phylotree are the morphotypes of the representative fungal endophytes per assigned clade.

We combined our fungal sequences with the sequences of close relatives available in NCBI GenBank to construct the ITS phylogenetic tree. The fungal endophytes from the coralloid roots of *C. debaoensis* and *C. fairylakea* from different population sites were represented by eight different fungal orders (as shown by different colors in the phylogenetic tree; Figure 3). Four orders, i.e., Hypocreales, Glomerellales, Xylariales and Diaporthales, represented the class Sordariomycetes; two orders, i.e., Eurotiales and Chaetothyriales, represented the Eurotiomycetes; a single fungal order Pleosporales represented the Dothideomycetes; and Polyporales represented the Agaricomycetes. Furthermore, in order to describe the phylogenetic placement of the fungal endophytes, we partitioned and marked the clades in the phylogenetic tree with letters that represent a total of sixteen clades. Eight clades were generated in Sordariomycetes (clade A–H), four clades represent the class Eurotiomycetes (clade I–L), three for Dothideomycetes (clade M–O) and one for Agaricomycetes (clade P) and the last clade represented the outgroup Mucoromycota (Figure 3). The cultural appearances of the fungal isolates were also provided in Figure 3 and labeled with the same letter as in the clade in the phylotree.

Further analysis of the tree showed that fungal endophytes in the coralloid roots belonged to at least 33 genera. Hypocreales is the most represented fungal order with 55 isolates (46% of the total isolates) and 10 genera. In clade A, six endophyte sequences (=6 isolates) were noted and were grouped to *Pochonia chlamydosporia* and *Verticillium* sp. Clade B has seven morphologically distinct isolates and clusters with *Trichoderma*, five of which showed a close relationship to *Trichoderma hamatum*, one with unidentified species of *Trichoderma* and one with *T*. *harzianum* (clade B). In the same clade (B), doubletons and singletons (fungal species with two or one isolate/s) clustered to *Purpureocillium lilacinum* and *Tolypocladium album* with 100% of bootstrapping. Nine endophyte sequences clustered in clade C, six of which are closely related to *Gliocladiopsis* sp., two sequences to *Ilyonectria* sp. and a single isolate recovered from natural habitat of *C. debaoensis* clustered with *Penicillifer diparietisporus* with 100% of bootstrapping. One of the clades with the highest number of sequences grouped with the genus *Fusarium* specifically to *F. oxysporum* (=12 isolates) and *F. solani* (=2 isolates) with 98% and 100% bootstrap support, respectively (clade D). Interestingly, various morphologically distinct isolates matched to *F. oxysporum* were observed in this study (see fungal morphotypes in Figure 3, clade D). The last clade (E) in the order Hypocreales is composed of ten sequences strongly clustered to *Clonostachys*, where half of the sequences joined the *C. rogersoniana* with 99% of bootstrapping and the other half to *C. rosea* with 100% of bootstrapping. Isolates from *C. rosea* were observed with both white-to-gray and orange colonies (see fungal cultural morphology in Figure 3, clade E).

In a different clade (F), six sequences formed the order Glomerellales in which three sequences from the present study showed close relationship to *Colletotrichum gloeosporioides*. The second highest number of isolates in Sordariomycetes was observed with fungal order Xylariales and represented by a single clade (clade G). At least eight species supported with morphologically distinct colonies (see fungal morphotypes in Figure 3, clade G) were composed of one to three isolates within the genera *Annulohypoxylon*, *Castanediella*, *Hypoxylon*, *Nemania*, *Nodulisporium*, *Pestalotiopsis* and *Xylaria* and were identified in this clade. In another clade (H) representing the order Diaporthales, three sequences clustered to *Diaporthe* and three sequences to *Phomopsis* with high bootstrap support. Interestingly, among the genera identified, *Penicillium* and its teleomorph *Talaromyces* in the order Eurotiales were the most represented with a total number of 24 isolates belonging to nine and four distinct species, respectively (see clade I–L and the corresponding morphotypes; Figure 3). In the same order, three isolates have been identified as closely related to three different *Aspergillus* spp. with 100% bootstrap values. A separate clade (L) consisted of two sequences displaying high relatedness (100% bootstrapping) to *Exophiala pisciphila*, which is a member of dark septate endophyte in the order Chaetothyriales. Furthermore, eleven isolates belonged to order Pleosporales in the class Dothideomycetes and represented three clades (M–O). The first clade in this order consists of five sequences in which two of these grouped to *Curvularia* spp. and a singleton grouped to *Phoma*. Two isolates showed high relatedness to multiple but closely related genera such as *Setophoma* and *Alternaria* and to an unidentified fungal strain (Th05; KY607737) from a root sample. Clade N is composed of five sequences, three of which showed similarities with 99% bootstrapping to *Microsphaeropsis arundinis* and two sequences to *Pseudopithomyces maydicus* with 100% bootstrap values. Clade O is composed of a singleton isolate grouped with the fungal species *Periconia macrospinosa*. Four sequences of Basidiomycetous fungi in order Polyporales (clade P) were exclusively recovered from the roots of *Cycas fairylakea*. Two of these sequences grouped with *Rigidoporus vinctus* and *Leiotrametes lactinea,* while two isolates strongly clustered with *Fomitopsis* cf *meliae.*

### 3.3. Diversity and Community Analysis of Fungal Endophytes

The findings of our study showed that both *C. debaoensis* and *C. fairylakea* shelters various fungal species. A comparative taxonomic and species diversity analyses between hosts and population sites was performed using different estimators, i.e., S/G ratio or Taxonomic Diversity Index (TDI), Shannon–Wiener (H’) and Fisher’s Alpha (FA) diversity indices. The highest endophytic community diversities and fungal richness were noted for *C. debaoensis* (H’ = 2.7, FA = 11.1) followed by *C. fairylakea* (H’ = 2.5, FA = 9.8), with their samples both collected from the botanical garden (Table 2). In fact, a comparable number of species were recorded in these samples, both with 15 genera and with 23 and 21 species, respectively. This was followed by *C. fairylakea* from natural habitats with diversity values of H’ = 2.2, FA = 9.8 and fungal composition of 18 species and 16 genera (Table 2). The least diverse fungal assemblage was hosted by *C. debaoensis* from their population sites in the Guangxi province with comparable results (natural habitat: H’ = 2.2, FA = 8.2; reintroduced: H’ = 2.2, FA = 8.0). Fifteen taxa and 13 genera were identified in the reintroduction site, while 14 species and 12 genera were identified in natural habitat (wild population). Furthermore, the differences on the S/G ratio or TDI values between population sites, except for the *C. debaoensis* from the botanical garden, were not statistically significant, revealing that the taxonomic diversities among population sites were the same.

**Figure 4:**
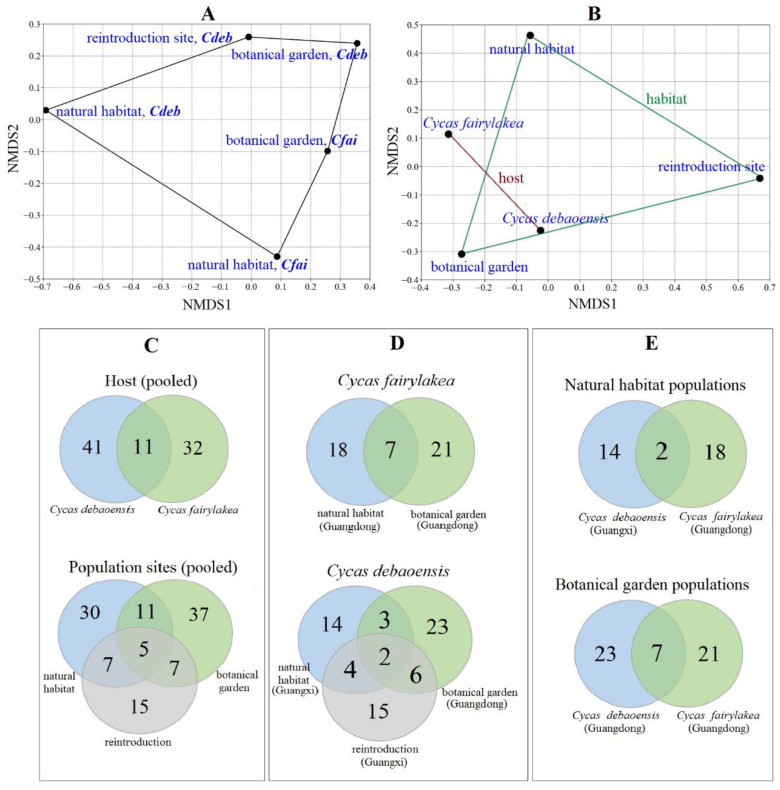
NMDS visualization based on Bray–Curtis analysis. The graphs showed the (**A**) ordination of the fungal communities between host species per population sites. (**B**) Plots are based on the pooled datasets of host *Cycas debaoensis* and *Cycas fairylakea*, regardless of the population sites and population sites, i.e., natural habitat, reintroduction site and botanical garden regardless of the host species. Venn diagrams illustrated the number of shared and unique taxa. Comparison was carried out between (**C**) pooled datasets from host species and population sites, (**D**) host species and (**E**) population sites over geographical region.

Ordination from NMDS revealed that the fungal endophytes in each host species from respective population sites showed unique and overlapping endophytic fungal communities (Figure 4A,B). A higher similarity was noticed in pooled datasets between the host species than population sites. This was statistically not supported by performing a non-parametric analysis of similarities (ANOSIM) which reflected no significant differences observed on fungal communities in between population sites (R = 0.5, *p* = 0.31) and host species (R = 0.08, *p* = 0.39). In addition, the analysis of similarities showed that the fungal community assemblage was also not significantly different (R = 0.42, *p* = 0.20) over the studied regional population sites, i.e., between the Guangdong and Guangxi provinces. Interestingly, a series of Venn diagrams highlighted the number of shared and unique fungal taxa between the host species and the regional and local population sites (Figure 4C–E). Only 11 species of fungal endophytes were present in both *Cycas* species, while only five taxa were shared between the three population sites (Figure 4C). This quantifies to a percentage of 17% and 8% shared species among host and population sites, respectively. Evaluation of fungal composition dissimilarities was also carried out for each host species per population site. Interestingly, seven species were shared between *C. fairylakea* collected from botanical garden and natural habitat from Guangdong province (Figure 4D). Only two species were common in *C. debaoensis* collected from the three population sites, four taxa were shared between reintroduction and natural habitat and both geographical sites were located in the Guangxi province (Figure 4D). Forty-five (73%) of the 62 fungal species were exclusively isolated from either host species in a single population site.

Taxonomic compositional similarities were also evaluated. *Talaromyces aculeatus* was the predominant and the only isolated fungal species which appeared in both host species across all population sites. The other shared fungal species were recorded in both hosts but not in all sites. For instance, *Gliocladiopsis* sp., *Verticillium* sp. and *Fusarium oxysporum* were recovered from four collecting sites except in *C. debaoensis* from the natural habitat. *Pochonia chlamydosporia* was absent in *C. fairylakea* from its natural population. Among the population sites, *Clonostachys rogersoniana* and *Diaporthe* spp. were exclusively recorded from both hosts in the botanical garden. Different species representing the genera *Nodulisporium* and *Phomopsis* were recovered only from the natural habitat of the two hosts. We also found a very limited number of taxa shared per population sites per geographical region. *Clonostachys rosea* was the only species common in Guangdong province and was not recorded in coralloid root samples from the Guangxi province. On the other hand, *Ilyonectria* sp. and *Pseudopithomyces maydicus* were exclusively reported in population sites in Guangxi. Others have been found in multiple sites but are exclusive to a specific host. For *Cycas debaoensis*, the exclusive species include *Exophiala pisciphila* and *Alternaria*/*Setophoma* spp. Fungal species exclusive to *C. fairylakea* were *Trichoderma* spp., *Fomitopsis* cf *meliae*, *Microsphaeropsis arundinis* and *Penicillium janthinellum*. In addition, all basidiomycetous fungi in this study were exclusively recovered from *C.* *fairylakea*. Other fungal taxa were recorded exclusively in one collection site. For example, the genera *Castanediella*, *Periconia* and *Tolypocladium* were unique in the reintroduction site of *C. debaoensis*. Similarly, the species representing *Penicillifer*, *Pestalotiopsis* and *Phoma* were found exclusively in the natural habitat of *C. debaoensis*, while *Annulohypoxylon* and *Leiotrametes* were unique in the natural habitat of *C. fairylakea*.

### 3.4. Screening of Antagonistic Potential Using Co-Cultivation Assay

**Figure 5:**
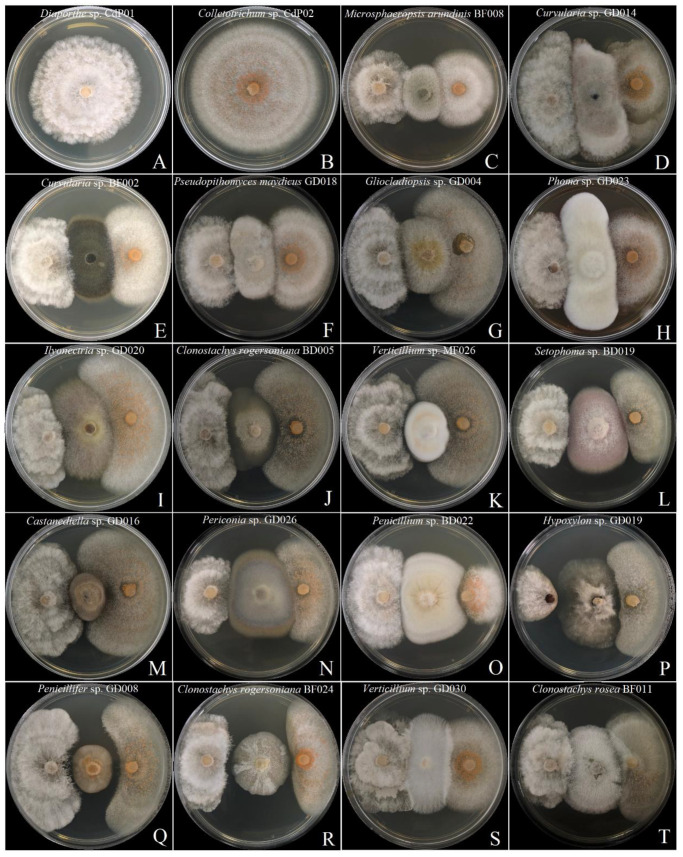
Results of the co-cultivation assay between nineteen fungal endophytes and the two fungal phytopathogens. (**A**,**B**) Control plates of the phytopathogens isolated from the diseased-symptomatic leaves of *Cycas debaoensis*. The two leaf fungal pathogens were co-cultivated with the fungal endophytes on PDA plates and incubated for 10 days at 26 ± 2 °C (**C**–**T**). (**C**–**I**) Displayed Type B interaction against the two pathogens; note that *Gliocladiopsis* GD004 displayed Type E interaction against *Colletotrichum* sp. (**G**). Type C interaction was observed in (**J**–**P**), while Type E and F interactions were observed in (**Q**–**T**) against the two phytopathogens.

One of the goals of our study was to discover fungal endophytes from endemic plant species with promising bioactivities. For this reason, the antagonistic activities of nineteen fungal endophyte isolates against the phytopathogens *Diaporthe* sp. CdP01 and *Colletotrichum* sp. CdP02 previously isolated from the diseased leaves of *C. debaoensis* were tested using the co-cultivation assay. The interactions between the fungal endophytes and the phytopathogens were characterized according to the criterion as described in our Materials and Methods Section. Results showed that six fungal species representing the genera *Curvularia*, *Microsphaeropsis*, *Ilyonectria*, *Phoma* and *Pseudopithomyces* showed type B interaction and mutually inhibited the two fungal phytopathogens (Figure 5, Table 3). In other cases, *Clonostachys rogersoniana* strain BD005 and *Gliocladiopsis* sp. showed Type B interaction against *Diaporthe* sp. but a different activity against *Colletotrichum* sp. In the separate co-cultivation plates of the two phytopathogens and the fungal strains, namely *Hypoxylon* sp., *Alternaria*/*Setophoma* spp., *Verticillium* sp. strain MF026, *Penicillium* sp., *Castanediella* sp. and *Periconia* sp., the fungal interaction type C has been observed (Figure 5). This interaction was characterized by inhibition of *Diaporthe* sp. and *Colletotrichum* sp. upon contact with the fungal endophytes where both phytopathogens continued to grow at a significantly reduced rate and the fungal endophytes (inhibitor species) grew also at a slightly reduced rate. Interestingly, several isolates showed promising activities and displayed Type E and F interactions. For instance, *Verticillium* sp. strain GD030 showed type E interaction and mutually inhibited the colony of the two phytopathogens upon contact but continued to grow at a reduced rate and advances over the colony of *Diaporthe* sp. The hypha of *Gliocladiopsis* sp. showed the same activity against *Colletotrichum* sp. Meanwhile, *Penicillifer* sp. and a different strain of *C. rogersoniana* BF024 inhibited the two phytopathogens at a distance while these isolates continued to grow at normal pace. In fact, a significant zone of inhibition (>2mm) was observed between these fungal endophytes and the phytopathogens. The fungal isolate *C. rosea* also manifested the type F interaction and further colonized the hyphal surface of the pathogens.

It is also interesting to note that pigment production was observed at the margin of some fungal isolates during physical confrontations with other isolates in the co-cultivation assay. For example, a dark pink pigmentation was observed at the margin between the colonies of *Alternaria*/*Setophoma* spp. and the phytopathogen, *Colletotrichum* sp. (Supplementary Figure S1). The hyphal portion of *M. arundinis* in contact with the phytopathogen *Diaporthe* sp. was also observed with black pigmentation after extending the assay incubation to 14 days (Supplementary Figure S1). On the contrary, the hypha of the phytopathogen *Diaporthe* sp. produced black pigmentation after its contact with fungal endophytes *Verticillium* sp. and *C. rosea* (Supplementary Figure S1). Meanwhile, the fungal endophyte species *Ilyonectria* sp. and *Phoma* sp. secreted an orange to red pigmentation onto the medium in its antagonism assay with the two phytopathogens.

The in vitro co-cultivation assay revealed that all 19 endophytic isolates inhibited the mycelial growth of the two pathogens, but the degree of inhibition varied per endophyte. The inhibition percentages (I%) of the fungal endophytes against *Diaporthe* sp. and *Colletotrichum* sp. were calculated with the results shown in Table 3. Fourteen (74%) and ten (53%) isolates showed 50–65% inhibition percentage against *Diaporthe* sp. and *Colletotrichum* sp., respectively. *Hypoxylon* sp. was recorded with the highest I% against *Diaporthe* sp. at 87.7%. This was followed by *Penicillifer* sp. and *C. rogersoniana* BF024 with I% of 69% and 66%, respectively. Meanwhile, the colonial growth of the *Colletotrichum* sp. was significantly inhibited at 80% inhibition percentage by *C. rogersoniana* (strain BF024), followed by *Penicillium* sp., *Penicillifer* sp. and *C*. *rosea*. In terms of fungal interactions, eight (42%) and six (32%) fungal endophytes exhibited type B interactions against *Diaporthe* sp. and *Colletotrichum* sp., respectively. Seven (37%) and eight (42%) exhibited type C. One fungal endophyte showed type E interaction against the two phytopathogens while three endophytes were recorded with type F interaction.

## 4. Discussion

The specialized coralloid roots of cycads appear to be hotspots for fungal communities as demonstrated in the recent studies employing next generation sequencing [37,38,39]. These studies also suggest that endemic *Cycas* spp. may harbor rare, specific or potentially novel fungal taxa which could be explored for bioprospecting and biocontrol of plant pathogens. The present study therefore employs a culture-dependent technique to study the diversity and molecular phylogeny of endophytic fungi in the specialized roots of two endemic *Cycas* host species in China. This technique has also been used to generate good collection of viable and culturable endophytic fungal communities, which is a requirement for any bioprospecting projects, and to test their ecological functions in the latter part of the experiments. Furthermore, the fungal communities associated with *C. debaoensis* from the wild and reintroduced populations in the province of Guangxi in China were also reported for the first time in this study.

The fungal endophytes associated with *C. debaoensis* and *C. fairylakea* were represented mostly by the phylum Ascomycota and comparatively with few taxa from Basidiomycota, corroborating the same results of previous studies on root-associated fungal endophyte communities [7,11,38,39,54]. In addition, Sordariomycetes was the most prevalent class of endophytic species in our study, followed by Eurotiomycetes, Dothideomycetes and Agaricomycetes as shown previously. Overall, a total of 62 species and 33 genera were molecularly identified from 30 plant individuals of the two *Cycas* hosts collected from different population sites. Previous study also reported 346 strains and 37 genera of fungal endophytes isolated from the endemic *C. panzhihuaensis* from Sichuan, China [54], and thus our study reflected the expected number of fungal genera in cycads. However, out of these only 58 strains and 16 genera were reported to have been isolated from the coralloid roots. In particular, the genera *Diaporthe*, *Aspergillus*, *Trichoderma*, *Alternaria*, *Curvularia* and *Fusarium* were previously reported in coralloid roots and were also identified in this study. Four other genera such as *Paecilomyces* (*Purpureocillium*), *Periconia*, *Phoma* and *Pestalotiopsis* were also identified in the present study but have been reported in other tissues, i.e., normal root, bulb, leaves and megasporophyll of *C. panzhihuaensis* [54]. Interestingly, about 70% of our reported fungal genera were not previously isolated, suggesting a taxonomically diverse fungal endophyte community recovered from the coralloid roots of the endemic *Cycas* species in Guangdong and Guangxi.

The genera *Talaromyces* and *Fusarium* that dominates the two *Cycas* spp. were common and ubiquitous in a wide range of plant host species [7,12,15,27,41,54]. As we might expect, these taxa were likely to be transmitted by the coralloid roots from the surrounding soil and house to its inner compartments as they have been frequently isolated in soil samples [14,51]. Interestingly, the high recurrence of fungal species *Talaromyces aculeatus, Fusarium oxysporum*, *Gliocladiopsis* sp. and *Pochonia* sp. across population sites may be attributed to the generalist status of these taxa or, as we have hypothesized, may also suggest their potential permanent association to cycads. In fact, *Fusarium* sp. was also among the single taxon of fungal endophytes that dominates the coralloid roots of *C. panzhihuaensis* from individuals in wild and botanical garden in the Sichuan Province [54]. The genera *Verticillium*, *Penicillium* and *Trichoderma* were also reported in the coralloid roots of *Cycas*. Fungal species under these genera were common saprophytes and pathogens or often colonized plant hosts as endophytes [12]. Interestingly, other genera, namely *Colletotrichum*, *Diaporthe* and *Phomopsis*, mostly inhabit above-ground organs [1,15,21,55], but were recorded from the coralloid roots. A possible migration of microbial associates within host tissues was reported in a few studies [15,56]. One such example is the detection of a high number of shared fungi in between above-ground and below-ground tissues in the exotic plant *Ageratina adenophora*, suggesting the potential transmission of fungal endophytes from air to leaves to stem to roots or vice versa [15]. The same pattern was observed among six forbs species but showed evident systematic growth of fungal endophytes through vertical transmission in seeds and recovered the same fungal taxa from the leaves [57]. Systemic growth of fungal endophytes in many plants remains poorly understood and, therefore, it would be particularly interesting to study whether the fungal endophytes associated to cycads were seed-transmitted, especially since the reproduction of these plants was primarily dependent on seed propagation. Alternatively, since the coralloid roots are sometimes found above the surface of the soil, it is also likely that some of the fungal endophytes commonly infecting the above-ground tissues via airborne fungal spores have non-systemically infected the coralloid roots similar to many foliar and stem endophytes [19,41,55], thus explaining the presence of some of the most common fungal communities thriving within the coralloid roots.

Rarely occurring taxa with singleton or doubletons isolates were also found to be members of fungal communities of the coralloid roots and exclusively found in one or two population sites. Particularly interesting are the rare taxa that were isolated in the limestone area in Guangxi. *Phoma* spp., which is a common root endophyte [13], has only a single isolate from *C. debaoensis* in the karst habitat. Furthermore, lesser known fungal genera, namely *Castanediella*, *Penicillifer*, *Pseudopithomyces*, *Ilyonectria* and *Periconia*, were only recovered from the population sites in Guangxi. Some of these genera have been previously reported in relatively few plants found in the same or nearby region, such as *Panax notoginseng* from Yunnan [58] and the wild *Dysosma versipellis* from Guangxi [28], indicating a potential biogeographical pattern of these fungal taxa. On the other hand, other taxa we recorded, such as *Pseudopithomyces*, were previously recovered from the aerial stilt root of mangrove (*Rhizophora apiculata*) [59] and the high abundances of *Periconia* were found in roots of grasses [60,61], suggesting its strong affinity to roots. Interestingly, *Penicillifer* has few new records recovered from soil samples in Bangladesh [62] and Korea [63]. To the best of our knowledge, reports on its association with plants, especially in roots, are very few in number and consists of mostly unpublished data. Its recovery from the roots of cycads along with other fungal endophytes could suggest their role to the hosts in adapting to the karst environment since many fungi provide service to plants, especially in unfavorable environments [6,9,37]. For example, some fungal endophytes may facilitate the adaptation of native *Colobanthus quitensis* to an environment with low temperature and extreme aridity [9]. Similarly, the presence of fungal endophytes in the special roots of cycads equally play a pivotal role that allow them to withstand the harsh environment and to further improve their growth, particularly in the condition of their habitat 300 million years ago [37,38].

**Figure 6:**
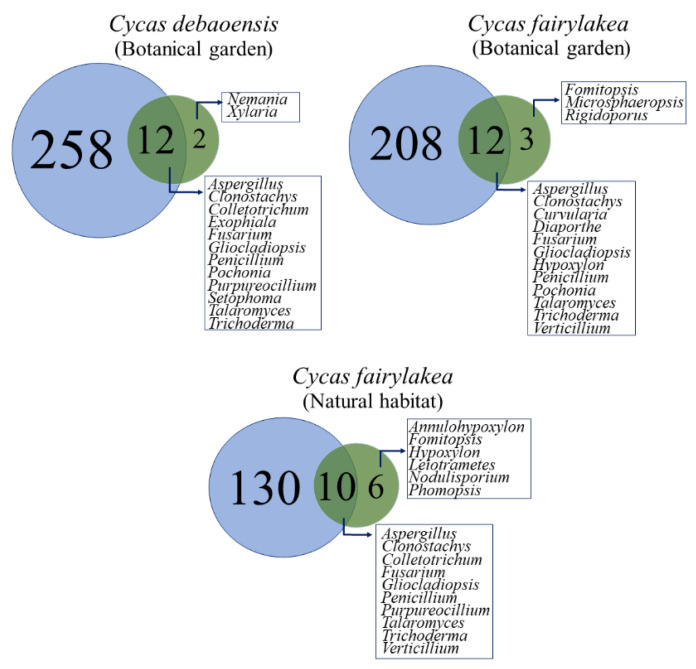
Venn diagrams expressing the number of shared and unique fungal endophytes between culture-independent (in **blue**) and culture-dependent (in **green**) methods. We listed the fungal genera recovered by both methods and those that were recovered solely by the culture-dependent method. The list of fungal taxa unique to culture-independent method is provided in Supplementary Table S2. The datasets based on the culture-independent method was obtained from the previous study in overlaps with some plants used in the present study collected from the same sites [39].

The differences in the fungal communities between host and sites were more distinctive when the taxonomic composition is considered compared to the diversity values, as the numbers are influenced by the biases brought by the traditional culture-based methods in investigating fungal diversity [15,44]. In fact, the present study only recovered a very small portion of the total fungal communities from the coralloid roots when compared to the high-throughput sequencing datasets in the earlier study carried out by the same authors, although the similarity of the fungal taxa that were isolated was high since some of the plants used in the present study came from the samples in the previous metagenome-based study [39]. Direct comparison reveals that 60–80% of the fungal genera reported here (Figure 6) were also detected with high relative abundance in the study with culture-independent approach (see Supplementary Table S2). However, the genera, namely *Cladosporium*, *Mycena*, *Scytalidium* and *Simplicillium*, were previously detected with high relative abundance but were not recovered using the culture-based approach. Similarly, several fungal endophytes, about 10–20% (Figure 6), were successfully isolated but were not detected in the previous study. The high-throughput sequencing may have possibly recovered these fungi but comparisons to databases have resulted in poor taxonomic information as was confirmed by the many OTUs recorded but were only assignable to a higher taxonomic ranking, e.g., class, order and family level [39]. Therefore, we highly suggest incorporating the two approaches (culture-dependent and culture-independent) when conducting biodiversity studies to obtain a complete and broader picture of the fungal endophyte community since these two different approaches complement the limitations of each other [64].

As clearly illustrated, the botanical garden samples have the highest number of isolates, community richness and diversity, while no significant difference was found between reintroduction site and natural habitat. However, the species estimator curves also did not reach the point of saturation (Figure 2A), indicating that more species are still expected for isolation. Several relevant factors including controlled growing conditions and differing habitat management may have initiated the increase in fungal assemblages in the botanical garden resulting in higher isolates as was observed with previous studies [41,44] and in our study. Neighboring plant richness in the botanical garden may equally contribute to this pattern as they can be donors of fungal propagules relative to one another [44]. It is also worth noting that samples from Guangdong province have more fungal endophytes yield than samples from the Guangxi province. The functional requirements and the local climatic conditions may have driven the assembly of endophytic fungi in plant tissues [15,16], which might also explain this result. It is worth recalling that Guangdong possesses humid and moist conditions (see Table 1 for reference) when compared to Guangxi, which has a relatively dry environment. The former conditions support fungal growth [15,16] and may have even promoted the germination of dormant spores that are typically inactive under natural dry habitat conditions [65], thereby resulting in the high fungal richness associated with the Guangdong samples. In addition, the Guangxi region with karst topology has been reported with extremely poor nitrogen and phosphorus elements and poor water conservation resulting in a dried-up soil surface [66]. Since fungal community is strongly driven by available phosphorus and water [67], these conditions may perhaps cause a decline in fungal biomass in the soil, which directly impacts the available inocula that may infect plant endosphere, such as roots, and therefore have supported our previous explanation as to why a lower richness was detected for Guangxi samples. However, this idea needs further studies as fungal endophytes, particularly the root-associated fungi, are often known to promote plant growth through the acquisition of minerals and would expectedly be advantageous in nutrient-poor habitats.

In addition, the Venn diagrams directly reflect the very small number of shared taxa between population sites suggesting that fungal endophyte communities in the coralloid roots of cycads may also be dependent on host-related factors as shown previously in other studies [41,44]. Host species identity and phylogenetic signals were reported to play a significant role on foliar and root associated endophytes [41,68,69]. An evidence showing the dissimilarity in phyllosphere fungal communities in *Mussaenda pubescens* was found to be positively correlated with the detected genetic differentiation among its regional populations [19]. Experiments on *Arabidopsis* revealed that host genotypes have little effects on the composition of its root endophytic microbes [68]. Similarly, a recent study showed that the host genotype was found to significantly contribute to the shaping of the root communities of fungal endophytes in wheat cultivars [69]. In Cycadaceae, the use of ISSR molecular markers in previous studies showed that the degree of genetic variation is high in between populations of the same species [33,34,35], indicating a clear plant genetic distance. Determining the genetic differences among the populations of *Cycas fairylakea* and *C. debaoensis* used in the present study can specifically determine the influence of hosts genotype on the assemblage of fungal endophytes in their coralloid roots and certainly merits future studies.

What is of interest is that our findings also highlight the possible influence of transplantation as we observed moderate varying microfungal communities between plants in the natural habitat and the grown seedlings that were planted in the botanical garden or reintroduction site. In fact, contrary to our predictions, the populations of *C. debaoensis* grown in the natural habitat and the populations for which the seeds were propagated, raised in nursery in a botanical garden for some time and then planted to the reintroduction site slightly differed in fungal composition, with only four shared taxa (28%). The similarity is comparatively low in spite of the fact that the reintroduction site was just 8 km away from the natural habitat and therefore was recorded to share the same environmental and climate conditions. Similarly, the *C. debaoensis* and *C. fairylakea* in the botanic garden, which we would expect otherwise, showed no significant difference in the values of their diversity and had only seven shared taxa (30%). We speculate that transplantation, including the time it was carried out, may have contributed to the changes of fungal communities associated to the *Cycas* host species across their population sites. The same pattern was observed in a previous study reporting different fungal communities between the roots of the grasses *Bouteloua* species raised in greenhouses and then transplanted to a natural field [70]. In support of this, a prior experimental study demonstrated that the transplanted plants required a certain period of time to be well-adapted in the new habitat where it takes at least six months to establish a distinct rhizosphere communities conformed from the resident soil communities [71,72]. Since soil, particularly the rhizosphere, is the imminent source of microfungal inocula for root infection, perhaps more time is needed to horizontally acquire it for their plant compartments, such as the roots. Therefore, this possibly explains our result that due to the time difference in introduction, the fungal communities in the *C. fairylakea* transplanted to the garden for 2.5 years from the time of sampling are distinct from those plants that were planted at longer period of time in the botanical garden, such as *C. debaoensis* which was planted for 16 years. This may also support the case of the individuals of *C. debaoensis* in Guangxi, which are at 12 years of post-transplantation to the reintroduction site and are displaying different fungal communities than compared to the natural habitat. The fungal endophytes present in their roots prior to transplantation were perhaps replaced by a different community acquired in the new habitat after long-time establishment. Our assumptions however require more experimental support due to the sample size, which limits our ability to draw further assessments. Certainly, experimental validation in which various experimental factors, e.g., the use of even-aged individuals, determination of the fungal communities on the nursery-grown cycads before and after transplantations must be considered for future studies.

We also conducted a co-cultivation assay to test the ability of fungal endophytes associated with *Cycas* spp. to be potential biocontrol agents of two phytopathogens *Colletotrichum* sp. and *Diaporthe* sp. The genera *Colletotrichum* and *Diaporthe* are important causative agents of anthracnose to many crops and a grapevine dieback disease, respectively [73,74]. Increasing evidence showed that fungal endophytes recovered from native or endemic plants may hold significant interest for their bioactive compound production and were shown to be useful in attacking pathogens [21,26,28,75]. For instance, three fungal endophytes from endemic plants in Central Andean Precordillera were found to secrete volatile compounds and suppressed the sporulation of filamentous necrotrophic fungus *Botrytis cinerea* [75]. Likewise, fungal isolates recovered from native Chilean gymnosperms also showed positive inhibition activity against several pathogens [25]. Specifically, *Verticillium* sp. isolated from roots of wild *Rehmannia glutinosa* showed strong antifungal activity against the pathogen *Pyricularia oryzae* [76]. In our study, the two *Cycas* spp. endemic to China harbored taxonomically distinct fungal communities in their coralloid roots that also can potentially produce a broad spectrum of secondary metabolites. In fact, the fungal interactions between the endophytes and the phytopathogens in the co-cultivation assay showed that some of the fungal endophytes are excellent candidates for bioprospecting. For example, fungal endophytes representing the genera *Periconia*, *Alternaria/Setophoma*, *Penicillium*, *Verticillium* and *Hypoxylon* out-grew the phytopathogens and restricted the growth of their mycelium. The mechanism possessed by these isolates may be similar to fungal endophytes with known antagonistic activity and inhibits the pathogens through competition, antibiosis and/or mycoparasitism [49,50,51,52]. In fact, some of the isolated genera, e.g., *Penicillium* and *Alternaria*, have been reported as potential biocontrol agents of several plant diseases [52,77]. In addition, previous studies revealed diverse compounds naturally recovered from *Periconia macrospinosa* and *Hypoxylon* spp., which potentially exhibited antimicrobial activities [78,79]. Interestingly, *Clonostachys rogersoniana* BF024, *C. rosea* and *Penicillifer* sp. were found to exhibit striking activities against the two phytopathogens (Figure 5). As was elucidated in the review, *Clonostachys rogersoniana* and *C. rosea* are known to produce a variety of compounds with known antibacterial, antinematodal, anti-dinoflagellate and cytotoxic activities [80,81,82]. In fact, *C. rosea* has been found effective against many kinds of fungal pathogens and the analysis of transcriptome data showed the presence of plenty of biocontrol genes [80,81]. No study has been reported specifically on the antifungal activity of *C. rogersoniana*. It is interesting because our study further demonstrated that the antagonistic activities of fungal endophytes from the coralloid roots are strain-specific, as exemplified by the different activities shown by *C. rogersoniana* strains BD005 and BF024 against the two pathogens. This species is known to produce gliocladiosin and rogersonin, which showed moderate antibacterial activity against *Klebsiella pneumoniae* and *Bacillus subtilis* [82], and could also be the same compounds responsible for the significant inhibition of strain BF024 against the two phytopathogens in our study. In addition, another interesting result was observed with a rare and slow-growing fungal species *Penicillifer diparietisporus*, which has the great potential to produce promising secondary metabolites as shown by the significant high inhibition percentage in the present study. What is of special interest is that this species also produces a distinct woody odor, indicating that it could be a lucrative source of bioactive compounds.

Some of the fungal isolates in a co-cultivation assay were observed with pigment productions on their hyphae suggesting that one fungus is releasing a compound to further impede the growth of the other. A similar observation was shown in the study of Hamzah et al. [52], where an endophytic fungus *Alternaria macrospora* secreted a yellow pigmentation on the medium during its contact with the pathogenic *Fusarium solani* since day three of the dual culture assay. Fungal pigmentations were reported to act as fungal armor and assisted fungi in sustaining adverse conditions or stressful environments [83]. In some experimental studies, fungal endophytes provided thermal protections to plants and demonstrated that ability to produce melanin pigmentation that possibly dissipates heat along the hyphae and/or complex with oxygen radicals generated during heat stress [84,85]. The production of pigmentations of some fungal endophytes in the co-cultivation assay was potentially a chemical defense for protecting their colonies against the other microbes, especially as a means of survival under the natural conditions of their host plant.

## 5. Conclusions

We revealed a phylogenetically and unique group of cultivable endophytic fungal communities colonizing the apogeotropic coralloid roots of the endemic *Cycas debaoensis* and *C. fairylakea*. The endophytic fungal assemblage was dissimilar between hosts species among population sites and were possibly influenced by a couple of factors, including local climatic conditions and age of transplantation. Our study further demonstrated that fungal endophytes from endemic hosts as in the case of our two *Cycas* spp. can be promising sources of bioactive compounds. In fact, our study tested, for the first time, the antagonistic activities of fungal endophytes recovered from the coralloid roots of Chinese *Cycas* spp. and our results showed that they may be considered as effective antagonists or biocontrol agents. Therefore, further investigation of their secondary metabolites, particularly for identifying and elucidating the compounds from those fungal isolates that show pronounced activities in co-cultivation assay, is needed. Our study fills the research gap by providing comprehensive data on the diversity and communities of cultivable fungal endophytes, including their possible ecological relationship to their host cycads.

## Figures and Tables

**Table 1:** Data on the host species and sample collection sites.

Host	Population Sites	Locality	GPS/Altitude	Habitat Description	Collection Date
*Cycas* *debaoensis*	botanical garden	^1^ NCGCC, FLBG, Shenzhen, Guangdong	22°34′36.1″ N 114°10′51.1″ E 60 ± 30 m.a.s.l.	Living collections of cycads in a controlled and managed environment; individuals used for the study were planted in 2004.	26 January 2020
	natural habitat	Debao County, Baise, Guangxi	23°29′29.2″ N 106°12′50.8″ E 905 ± 24 m.a.s.l.	Dispersed in open landscapes and on limestone hills with shrubs and trees; some cycad plants growing on cracks or in thin soil near the edges of limestone.	22 October 2020
	reintroduction site	^1^ Huanglian mountain nature reserve, Debao County, Baise, Guangxi	23°33′47.1″ N 106°14′09.8″ E 885 ± 8 m.a.s.l.	Scattered on open slopes with grasses and sparsely covered by shrubs and small trees; local population was reintroduced in 2008.	22 October 2020
*Cycas fairylakea*	Botanical garden	^1^ NCGCC, FLBG, Shenzhen, Guangdong	22°34′36.4″ N 114°10′50.6″ E 55 ± 20 m.a.s.l.	Living collections of cycads in controlled and managed environment; individuals used for the study were planted in 2017.	26 January 2020
	natural habitat	Meilin Reservoir Park, Shenzhen, Guangdong	22°34′38.5″ N 114°00′34.2″ E 114 ± 23 m.a.s.l.	Growing on slopes of ridges and cliffs along river valleys and understory of moist closed subtropical forests at low elevations.	21 January 2020

^1^ National Cycad Germplasm Conservation Center (NCGCC); Fairy Lake Botanical Garden (FLBG).

**Table 2:** Records and diversity of fungal endophytes associated with the hosts *Cycas* spp. from different population sites. Values followed by the same letter are not significantly different, *p* < 0.05.

^1^ Sample Source	^2^ Records of Fungal Endophytes							
	R	G	S	S/G Ratio	H’	FA	CR (%)	IF (%)
*Cdeb*, Nh	37	12	14	1.2 ^a^	2.2 ^a^	8.2 ^a^	17	13
*Cfai*, Nh	52	16	18	1.1 ^a^	2.2 ^a^	9.8 ^b^	24	18
*Cdeb*, Bg	77	15	23	1.5 ^b^	2.7 ^b,c^	11.1 ^c^	36	27
*Cfai*, Bg	74	15	21	1.4 ^a,b^	2.5 ^c^	9.8 ^b^	34	26
*Cdeb*, Re	44	13	15	1.2 ^a^	2.2 ^a^	8.0 ^a^	20	15
*Cdeb* (Pooled)	158	29	41	1.4 ^a^	3.0 ^a^	17.8 ^a^	24	56
*Cfai* (Pooled)	126	21	32	1.5 ^a^	2.7 ^b^	13.8 ^b^	29	44

^1^ Host: *Cdeb*: *Cycas debaoensis; Cfai*: *Cycas fairylakea*; Population site: Nh: natural habitat; Bg: botanical garden; Re: reintroduction site. ^2^ Records of Fungal Endophytes: R = individuals/records, G = number of genera, S = number of species, H’ = Shannon diversity index, FA = Fisher’s Alpha diversity index, CR = colonization rate, IF = isolation frequency.

**Table 3:** Results of the in vitro co-cultivation assay of nineteen selected endophytes against the two phytopathogens. The type of fungal interaction for each isolate against each pathogen and the percentage of growth inhibition (I%) ± standard deviation (SD) was indicated. See Figure 3 for the description of the clades.

Species Name and Isolate Code	Clade	*Diaporthe* sp. Strain CdP01		*Colletotrichum* sp. Strain CdP02	
		Percent Inhibition (I%) ± SD	Type of Interaction	Percent Inhibition (I%) ± SD	Type of Interaction
*Castanediella* sp. GD016	G	58.7 ± 1.89	C	63.3 ± 1.57	C
*Clonostachys rogersoniana* BD005	E	52 ± 1.63	B	62.2 ± 1.50	C
*Clonostachys rogersoniana* BF024	E	66.7 ± 1.89	F	82.1 ± 1.50	F
*Clonostachys rosea* BF011	E	64.3 ± 2.05	F	80 ± 1.63	F
*Curvularia* sp. BF002	M	50 ± 1.63	B	56.7 ± 2.72	B
*Curvularia* sp. GD014	M	51.6 ± 0.47	B	53.9 ± 2.1	B
*Gliocladiopsis* sp. GD004	C	56 ± 3.27	B	68.3 ± 1.36	E
*Hypoxylon* sp. GD019	G	87.33 ± 0.94	C	70.3 ± 1.89	C
*Ilyonectria* sp. GD020	C	60 ± 3.77	B	61.7 ± 1.70	B
*Microsphaeropsis arundinis* BF008	N	42.7 ± 3.77	B	51.1 ± 2.83	B
*Penicillifer* sp. GD008	C	69.3 ± 3.30	F	81.1 ± 3.14	F
*Penicillium* sp. BD022	J	64 ± 2.83	C	81.5 ± 2.51	C
*Periconia* sp. GD026	O	62 ± 2.83	C	69.3 ± 1.89	C
*Phoma* sp. GD023	M	61. 3 ± 4.99	B	65.6 ± 4.16	B
*Pseudopithomyces maydicus* GD018	N	58.7 ± 1.89	B	65.6 ± 1.57	B
*Alternaria/Setophoma* sp. GD005	M	61.3 ± 1.89	C	75.6 ± 1.57	C
*Alternaria/Setophoma* sp. BD019	M	62. 3 ± 0.94	C	71.1 ± 2.83	C
*Verticillium* sp. MF026	A	53.3 ± 1.89	C	65.56 ± 1.57	C
*Verticillium* sp. GD030	A	49.3 ± 1.89	E	58 ± 2.79	E

## Data Availability

All sequences generated in this study were submitted to GenBank. Other data presented in this study are available in Supplementary Materials here.

## Supplementary Materials

The following are available online at https://www.mdpi.com/article/10.3390/jof7070572/s1, Figure S1: Reverse plates illustrating the results of the in vitro co-cultivation assay between the fungal endophytes and phytopathogens; Table S1: BLAST analysis showing the maximum nucleotide identity of representative fungal isolates based on ITS rDNA sequences; Table S2: List of fungal taxa recovered by culture-independent method, the datasets were extracted from the study of Pecundo et al. [39].
