# Supplementary figures and images for: Diversity, Phylogeny and Antagonistic Activity of Fungal Endophytes Associated with Endemic Species of Cycas (Cycadales) in China
Source: J Fungi (Basel). 2021 Jul 18;7(7):572. doi: 10.3390/jof7070572 (PMC8304459; doi:10.3390/jof7070572)

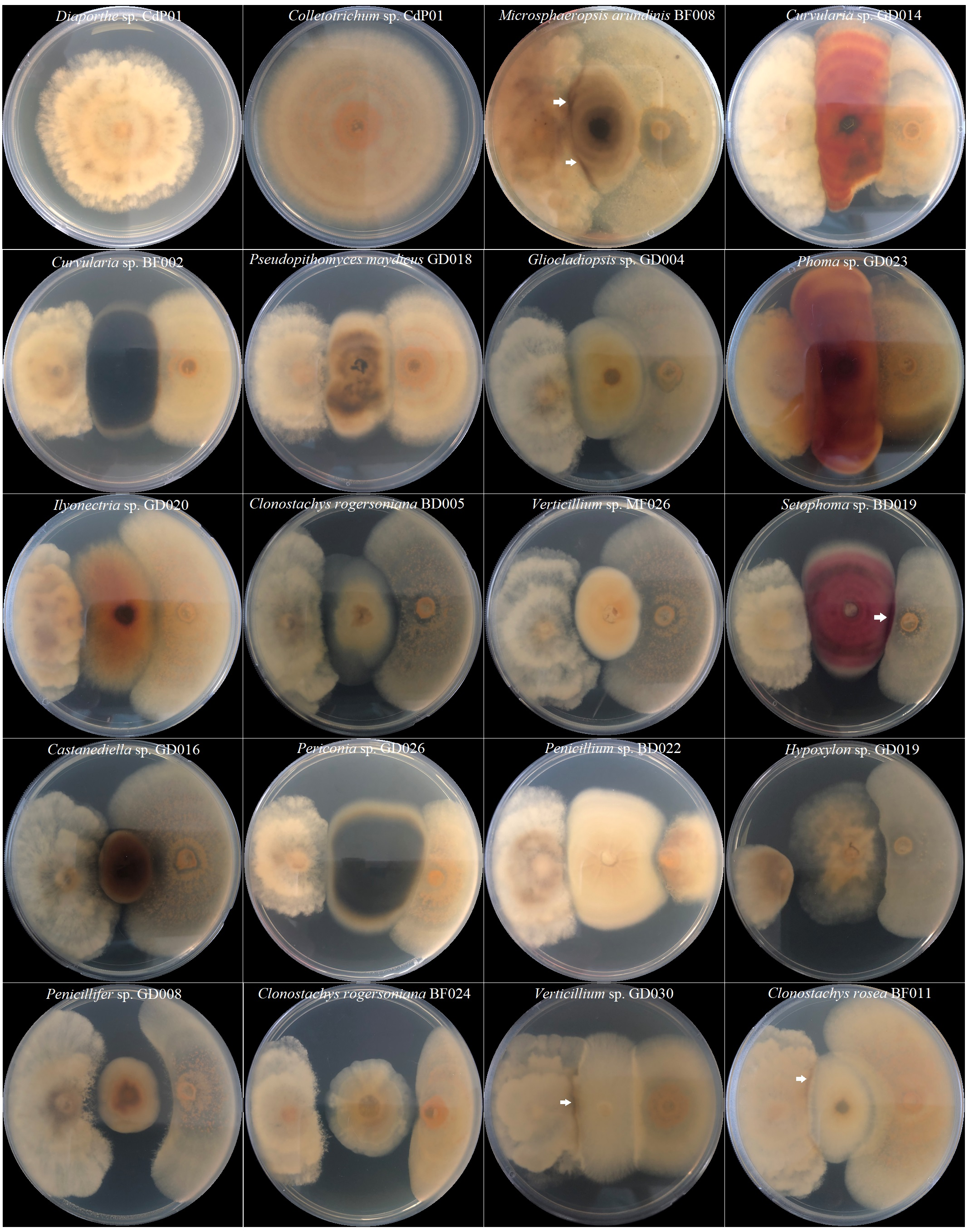

Supplement: Supplementary file 1 [file jof-07-00572-s001.zip › Supplementary Figure S1_Reverse plates_Fungal interactions.tif]
